# Influence of Freeze- and Spray Drying with Carrier Agents on Alkamides, Antioxidant Properties, and Process Contaminants in *Echinacea purpurea* Root Extract Powders

**DOI:** 10.3390/molecules30193864

**Published:** 2025-09-24

**Authors:** Mariusz Kułaga, Klaudia Masztalerz, Jessica Brzezowska, Anna Michalska-Ciechanowska

**Affiliations:** 1 Laboratorium Analiz Chemicznych Spark-Lab Sp. z o.o., Al. Zwycięstwa 96/98, 81-451 Gdynia, Poland; 2 Department of Fruit, Vegetable and Plant Nutraceutical Technology, Faculty of Biotechnology and Food Science, Wrocław University of Environmental and Life Sciences, Chełmońskiego 37 Str., 51-630 Wrocław, Poland; jessica.brzezowska@upwr.edu.pl; 3 Institute of Agricultural Engineering, Faculty of Life Science and Technology, Wrocław University of Environmental and Life Sciences, Chełmońskiego 37A Str., 51-630 Wrocław, Poland; klaudia.masztalerz@upwr.edu.pl

**Keywords:** *Echinacea purpurea* (L.) Moench, powders, maltodextrin, pea protein isolate, carrier blend, spray drying, alkamides, antioxidant capacity

## Abstract

*Echinacea purpurea* (L.) Moench root is a rich source of alkamides and other bioactive compounds with potential health-promoting effects. This study aimed to evaluate the influence of drying technique and carrier type on alkamide content, antioxidant properties, and process contaminants in *E. purpurea* powders. Root extracts were subjected to freeze-drying or spray drying at air inlet temperatures of 150, 170, and 190 °C, with maltodextrin, pea protein isolate, or their blend used as carrier agents. The resulting powders were analyzed for physical and chemical properties, including alkamides concentration, total phenolics content, antioxidant capacity, free amino group levels, and markers of advanced Maillard reaction products. Spray-dried powders had a moisture content lower than 2.3%, compared with an average of 7.7% in freeze-dried samples. Spray drying at 150 and 170 °C combined with the maltodextrin–pea protein blend resulted in the highest alkamide levels, while total phenolics content and antioxidant capacity were retained at levels comparable to freeze-drying. Neither hydroxymethyl-*L*-furfural nor furfural was detected via HPLC in any sample. Overall, spray drying under the tested conditions represents a favorable alternative to freeze drying, yielding *E. purpurea* root extracts powders with higher alkamides content, similar antioxidant properties, and absence of process contaminants.

## 1. Introduction

*Echinacea purpurea* (L.) Moench (*E. purpurea*) is a plant from the *Asteraceae* family. It is commonly called purple coneflower because of its characteristic cone-shaped inflorescence. *E. purpurea* is mainly known for its polysaccharides, glycoproteins, caffeic acid derivatives and alkamides [1]. Such a wide spectrum of bioactive compounds is responsible for its immunostimulatory [2], antioxidant [3], antibacterial [4] and anti-inflammatory [5] properties. *E. purpurea* was used by indigenous people as a pain reliever and a medicine for infections and inflammations [6]. Due to its numerous scientifically proven health benefits, *E. purpurea* has been widely incorporated into various health-related products. To date, extracts of *E. purpurea* are available on the market in the form of capsules, tablets, poultices, tinctures, teas and others. Furthermore, the literature also reports enrichment of juices [7,8], nectars [9], meat [10], cakes [11], and fruits with basil seed gum [12] containing *E. purpurea* extract. Building on these applications, we propose a novel, convenient and easily applicable form, i.e., *E. purpurea* extract powders, as a dietary supplement or a functional food ingredient. This powdered form has the potential to facilitate broader and more flexible usage in the food sectors. The global market for *Echinacea* extract-based products is experiencing significant growth, with Verified Market Reports projecting it to reach USD 150 billion in 2024 [13]. This dynamic market expansion highlights the substantial potential of developing new and consumer-friendly forms of *E. purpurea* extract, such as powdered format proposed in the study.

The health benefits of foods, including their ability to reduce the risk of chronic diseases, largely depend on their content of biologically active compounds. Thus, there is a need to search for stable forms of *Echinacea* that will increase the chances of delivering its bioactives to the human body in easy-to-handle form. Extraction is a common method for preserving desirable compounds from plants. However, liquid extract is an intermediate form of processing plant materials because it provides a potential favorable environment for microbial growth, enzymatic reactions, and active ingredient degradation [14]. Converting extracts into powders reduces the risk of losing bioactive compounds. In the food industry, spray drying and freeze-drying are commonly used to produce plant extracts in powdered form. The principle of spray drying is based on the formation of an aerosol and evaporation of liquid from fine droplets at elevated temperature, which may cause degradation of particular components [15]. In contrast, freeze-drying involves freezing the food material and subsequently removing the frozen water through sublimation under low pressure, yielding a powder. The use of high vacuum and freezing of extracts is associated with high energy demand during processing into powders [16]. Spray drying is considered a more economical and scalable method compared to freeze-drying, with reported expenses being roughly 4–8 times lower per kilogram of water removed [17] and in some large-scale industrial contexts, even 30–50 times lower [18]. Consequently, when no other constraining factors are present—such as the degradation of bioactive constituents or adverse changes in physicochemical characteristics—spray drying may be regarded as the more cost-efficient alternative.

Additionally, to improve the drying yield and limit the loss of bioactives from plant extracts during processing, carriers are used to enable the transformation of liquid extract to stable form of powder [19]. Carriers are food grade materials with the ability to create a protective layer on the outer surface of the powder particles [20]. Maltodextrin (M) is a starch-derived polysaccharide obtained by partial hydrolysis, typically carried out with the aid of acids or enzymes [21]. High solubility in water, neutral taste and the ability to reduce the viscosity of solutions make maltodextrin one of the most frequently used carriers in the food industry [22]. Proteins are increasingly being used as new types of drying agents due to their ability to create a porous and thick protective coating [23]. Among proteins, pea protein, often in a form of isolate (PPI), is used for drying of plant extracts because they are characterized by a relatively high nutritional value and lower allergenic potential [24].

The use of carriers may help to reduce the loss of bioactives, but degradation can still take place during the processing. Due to elevated temperatures of food processing, the non-enzymatic glycation reactions also called Maillard reaction may occur, i.e., reactions between amino acids, peptides or proteins and reducing sugars resulting in the formation of a wide range of brownish aromatic substances usually at the final stage [25]. The course of the Maillard reaction is affected by pH, water activity, time and temperature of heat treatment, concentration of reducing sugars, free amino acids, and therefore strictly depends on the composition of the food [26]. Changes in the matrix of processed food due to the Maillard reaction can be detected by analyzing loss of free amino groups and the content of reaction indicators, such as furfural and 5-hydroxymethyl-*L*-furfural [27].

Despite the wide range of products on the market enriched with purple coneflower extracts, there is a lack of studies on drying of root extracts. The hypothesis was that spray-dried (SD) powders formulated with maltodextrin and pea protein isolate, when applied to *E. purpurea* root extract, would retain bioactive compound levels similar to those found in freeze-dried (FD) counterparts. Therefore, the study aimed to fill this knowledge gap by evaluating the effects of carrier type and spray-drying temperature on the physicochemical properties of *Echinacea purpurea* root extract powders, with particular emphasis on alkamides content and compounds with antioxidant potential. In view of the above, maltodextrin, pea protein isolate, and their blend were selected as carriers due to their proven ability to enhance microencapsulation efficiency and protect thermolabile bioactive compounds during spray drying, as confirmed by previous studies [28,29,30]. This work provides novel insights into the preservation of bioactive constituents during drying of *E. purpurea* and supports the development of powders enriched in alkamides. In addition, the contents of furfural and 5-hydroxymethyl-*L*-furfural were assessed as markers of Maillard reactions and as food safety indicators.

## 2. Results and Discussion

### 2.1. Moisture Content (Mc)

In the case of control samples (no carrier addition), SD powders had on average 10 times lower values of *Mc* than FD samples, regardless of drying temperature applied for spray drying, which can enhance the stability of powdered products [31]. A significantly higher *Mc* in FD powders was also observed by Darniadi et al. [32] and Pellicer et al. [33] compared to SD powders. *Mc* of SD control powders showed that drying temperature in the range of 150–190 °C of *E. purpurea* root extract did not significantly differentiate the samples. This lower moisture content in SD powders can be attributed to the rapid water removal during atomization and contact with hot air, which efficiently evaporates water from both the surface and the particle core [34]. In contrast, freeze-drying relies on slower sublimation under vacuum, often leaving higher residual moisture [35].

When considering samples with added carriers, *Mc* results indicated that FD powders had significantly higher values than all SD powders (Table 1). Therefore, producing powders from *E. purpurea* root by spray drying may potentially reduce caking compared to freeze-drying due to the lower *Mc* [36]. The average *Mc* results for SD carrier-added powders obtained at 190 °C had more than 3 times lower *Mc* than powders dried at 150 and 170 °C [37,38]. Thus, it can be inferred that elevating inlet air temperature of spray drying enhanced the efficiency of water removal. The greatest impact of increasing the temperature to 190 °C was observed in the powders with the addition of M and blend composed of maltodextrin and pea protein isolate (M+PPI). These observations are consistent with Gong et al. [39] during drying of strawberry extract. Higher air inlet temperature during SD could promote the formation of a more compact microcapsule shell in powders with the addition of PPI or blend composed of M+PPI compared to M [40]. When the effect of carrier type was compared, it was indicated that the use of PPI or M+PPI resulted in products with lower *Mc* than M. In the group of FD samples, the carrier did not significantly differentiate the powders in regard to *Mc* (Table 1).

### 2.2. Water Activity (a_w_)

The *a_w_* values ranged from 0.0345 to 0.1551 (Table 1), which potentially increase the level of microbiological safety by inhibiting the growth of pathogens and spoilage microorganisms [41]. Among control samples, the SD powders had significantly lower *a_w_* than the FD products. The *a_w_* results also indicated that an increase in the SD temperature in the range of 150–190 °C effectively reduced the *a_w_* of the powders. In a comparison of carrier-added samples with respect to the drying technique, FD powders had significantly lower *a_w_* values when compared to SD, regardless of the inlet air temperature applied. An increase in SD air inlet temperature did not result in a significant reduction in the *a_w_* of powders derived from *E. purpurea* root extract. Regarding the effect of carrier type, powders with PPI exhibited, on average, 28% and 31% higher water activity than those with M and M+PPI, respectively. The superiority of M and M+PPI carriers among SD powders was further confirmed by significantly lower *a_w_*, regardless of drying temperature. On the other hand, FD powders did not differ significantly with respect to *a_w_* due to the type of carrier used.

In general, there was no significant correlation between the *a_w_* values and *Mc* (*r* = 0.27, *p* < 0.05) in *E. purpurea* root extract powders. Although FD powders exhibited the highest *Mc*, they showed the lowest *a_w_*, suggesting that water was strongly bound within the matrix, thereby limiting its availability for chemical degradation and potential microbial proliferation. The distinct principles underlying freeze-drying and spray drying lead to differences in microcapsule structure and powder composition, which in turn result in varying degrees of water binding [42].

### 2.3. Bulk Density

Bulk density is a parameter that influences the packaging of the product and its transport. The bulk density of controls did not differ, regardless of the drying technique used for their obtainment. Increasing the inlet air temperature reduced bulk density in SD control samples. A similar observation was drawn by Laokuldilok et al. [43] for black glutinous rice extract powders. The comparison of bulk densities in samples with added carriers indicated that the drying technique had no significant impact on this parameter. The increase in the SD drying temperature caused a significant decrease in the bulk density only in the case of powders with the addition of M. As previously observed, the samples produced by SD at 150 °C with addition of M (SD-150-M) had a higher *Mc* compared to the products gained by SD at 170 °C and at 190 °C with addition of the same carrier, which could be the reason for these differences. The addition of PPI alone resulted in powders with bulk density values that were, on average, 56% and 68% higher than those observed for M+PPI and M formulations, respectively. Therefore, the addition of PPI may resulted in a different particle size, morphology and degree of powder agglomeration, as these features determine the higher bulk density than M and M+PPI [39].

### 2.4. True Density

True density is a key physical property that influences the behavior of powders during processing. It plays a crucial role in operations such as mixing and granulation, affecting the homogeneity of the final product, especially in the powdered form. Based on the true density results (Table 1) for the control samples, it was concluded that neither the drying technique nor the air inlet temperature in spray drying significantly influenced this parameter. For samples with added carriers, the true density results indicated that spray drying with the addition of M produced powders with significantly lower true density. In turn, when comparing powders with the addition of PPI and M+PPI across drying techniques applied, no significant differences were observed. However, when analyzing the effect of the carrier on true density, the addition of M resulted in SD powders with an approximately 8% higher true density on average compared to those with PPI.

### 2.5. Porosity

The porosity of all controls did not differ significantly, indicating that the drying technique and parameters applied were not of major importance. In the carrier-added powders, the drying technique affected the porosity of the products differently depending on the type of added carrier. The porosity of the powders with the addition of PPI did not depend on the drying technique, while the SD-170 and SD-190 powders with the addition of M had higher porosity than FD-M, on average, by 15%. An increase in the spray drying air inlet temperature from 150 to 170 or 190 °C increased the porosity only in M-added powders, with no effect observed for PPI or M+PPI samples. Powders with the addition of PPI were characterized by an average porosity lower by 20% compared to M and M+PPI [44,45]. Therefore, the half-share of maltodextrin in the blend resulted in porosity similar to that of maltodextrin alone. In general, the use of maltodextrin or a blend of maltodextrin with pea protein isolate for drying of the *E. purpurea* root extract can significantly increase the porosity, which may potentially result in better retention of bioactive compounds [46,47].

### 2.6. Color and Browning Index (BI)

The color of the powders can affect the overall appearance of the food products, which can potentially be modified with *E. purpurea* root extract powders when added. Since visual appearance often determines a product’s attractiveness and consumer acceptance, the color of the resulting powders was evaluated in this study.

Among the controls, the lightness (L*) values were higher for SD powders, on average, when compared to the FD powders, which resulted in a brighter color (Table 2). It was also observed that L* were inversely correlated (*r* = −0.72, *p* < 0.05) with *Mc* of powders, which could be a potential reason for the differences in L* of SD and FD powders. Increasing the drying temperature of the controls during spray drying did not result in a consistent trend in L* values. When the powders with added carriers were compared, the same conclusion as for the control was drawn, i.e., SD powders were brighter than FD. The SD air inlet temperature was not a differentiating factor for the carrier-added samples, which could indicate a lack of reaction resulting in darker components at elevated temperatures. Evaluation of the L* parameter according to the carrier type demonstrated that powders formulated with PPI were considerably darker than those containing M or a combination of M and PPI [48].

Comparison of redness (a*) values of the control samples showed that the FD powders had higher values than SD powders, independent of the drying temperature, which resulted in a more reddish color of the FD powders. The drying temperature did not significantly differentiate the controls in the range 150–190 °C. When samples with added carriers were considered, a* results revealed that FD samples also had a redder color compared to SD, and that the drying temperature was not significant in the range tested. Similarly, as with the L* analysis, the addition of PPI resulted in a significant higher a* compared to samples with M and M+PPI. Comparing the results of a* and *Mc*, it was concluded that these parameters were correlated (*r* = 0.70, *p* < 0.05) and therefore *Mc* could be a factor determining a* of the *E. purpurea* extract powders.

The yellowness (b*) results showed that SD-150 powder had a significantly lower value among the controls, resulting in a less intense shade of yellow. Among the carrier-added samples, SD powders with PPI addition showed a more yellow shade compared to FD powder, while the opposite conclusion was drawn for maltodextrin-added powders. When SD powders obtained at different temperatures were considered, no statistically significant differences in b* values were noted. Within the SD powder group, it was observed that powders with PPI consistently showed higher coordinate b* values than powders with M or M+PPI, resulting in a more yellow shade of powders with PPI.

Comparison of the browning index (BI) values of powders can show potential alterations of matrix components that occurred during processing, especially when different carrier types are used for drying of plant-based extracts [49]. The BI of the controls indicated that SD powders had lower values than FD powders, which was associated with correlations with a* (*r* = 0.98, *p* < 0.05) reflecting the red-green color components and b* (*r* = 0.79, *p* < 0.05) determining the presence of yellow pigments. The drying temperature in the tested range did not result in significant changes in the BI of the SD powders. In the group of powders with the addition of carriers to liquid feed, the BI values indicated that all SD powders with the addition of M and M+PPI had lower BI values compared to FD powders. On the other hand, SD and FD samples with the addition of PPI did not show any differences. The comparison of BI results with respect to drying temperature concluded that the increase in temperature in the range of 150 to 190 °C does not cause changes in BI. Analysis of the type of carrier on BI indicated that the use of PPI gives powders with the highest proportion of brown pigments compared to M or M+PPI suggesting the possible progress of Maillard reaction toward browning due to the higher presence of proteins in liquid feed compositions submitted to drying.

### 2.7. Alkamides Content

In the study, six alkamides were identified and quantified in analyzed powders. The average concentrations of these components in samples from *E. purpurea* root extracts were 8.56%, 30.78%, 34.89%, 1.93%, 10.46%, 13.39% for alkamides 1–6, respectively. The concentrations of single alkamides in the powders (Table 3) strongly correlated with the sum of six alkamides (*r* ≥ 0.91, *p* < 0.05), which indicated a proportional effect of changes in processing parameters on the concentration of single alkamides in powdered products. The comparable influence of parameters and drying techniques on the concentration of specific alkamides is likely due to their similar chemical structures, which determine the same physicochemical characteristics [50]. Although alkamides contain multiple unsaturated bonds, which may increase susceptibility to thermal degradation in the presence of oxygen [51], despite differences in the arrangement of these unsaturated chains, the presence of phenolic compounds in the extracts can enhance their stability by protecting these bonds from oxidation [52]. This is likely one of the factors contributing to the strong correlation observed between total and individual alkamides content during drying. When the control samples were considered, it was observed that FD and SD powders dried at 150 °C and 170 °C had a higher alkamides content by 58%, 39% and 39%, respectively compared to SD powder dried 190 °C. Similarly, during drying of *Echinacea angustifolia* DC roots [53] and *E. purpurea* [54], temperatures up to 70 °C did not result in alkamide degradation. Therefore, it was concluded that spray drying may be an alternative to the more expensive and technologically more demanding freeze-drying with respect to the alkamides presence and content in powders. The results obtained for the controls also showed that an increase in temperature above 170 °C could result in degradation of these constituents. In the carrier-added samples, the SD powders had a 29% higher content, on average, compared to the FD powders, so the use of carriers effectively protects against SD conditions. A comparison of the alkamides contents in the powders across different spray drying air inlet temperatures indicated that a significant loss occurred only for the M+PPI-added samples when the temperature increased from 150 to 190 °C. M, PPI as well as their blend were effective agents for protection against elevated SD process temperature. For SD powders, a significantly higher alkamide content was determined in powders with M+PPI. This effect can be attributed to the complementary roles of M and PPI during spray drying. M acts as a glass-forming carrier that rapidly solidifies into a protective matrix, reducing oxygen and heat penetration into the particle core [40]. PPI, in turn, provides interfacial stabilization of the droplets and forms a protein film around the microencapsulated material, which further limits thermal degradation and volatilization of alkamides [55].

### 2.8. Total Phenolics Content (TPC)

Controls did not differ significantly with respect to the drying technique or the spray drying parameters applied (Table 4). However, there are studies reporting a decrease in phenolics content during high-temperature drying, both in the aerial parts of *E. purpurea* [54,56] and in the roots of *Echinacea angustifolia* DC [53]. These differences can be explained by the duration of exposure to elevated temperatures. In spray drying of extracts, the contact with high temperature is relatively short, whereas drying of whole plant materials may last several hours, leading to greater degradation of phenolic compounds and accounts for the seemingly contradictory findings. In carrier-added samples, the TPC remained unaffected by both the drying technique and the spray drying inlet temperature, with no statistically significant variations observed. Spray drying can therefore be regarded as a less expensive processing option when compared to freeze-drying [57]. The analysis of TPC results based on carrier type indicated that PPI provided less effective protection of phenolic compounds, as the average TPC values in powders containing PPI were 26% and 12% lower compared to those with M and M+PPI, respectively. TPC results did not correlate with alkamide content; therefore, the effect of processing *E. purpurea* extracts on these groups of compounds should be monitored individually.

### 2.9. Antioxidant Capacity In Vitro

Similarly to TPC results, among the control samples, no differences in the antioxidant capacity were observed due to the drying technique and SD air inlet temperature (Table 4). In group of the carrier-added samples, variations in drying technique and SD air inlet temperature showed no notable impact on the antioxidant capacity measured by TEAC ABTS and FRAP methods. In turn, the type of carrier influenced the antioxidant capacity, as significantly lower values were determined for FD-PPI and SD-170-PPI powders compared to the powders with the addition of M or M+PPI. The antioxidant capacity measured by TEAC ABTS (*r* = 0.97, *p* < 0.05) and FRAP method (*r* = 0.96, *p* < 0.05) showed a strong correlation with the TPC results; thus, higher TPC was associated with higher antioxidant capacity of *E. purpurea* root powders [58,59].

### 2.10. Furfural (FF) and 5-Hydroxymethyl-L-furfural (5-HMF) Content

The powders were assayed for the content of FF and 5-HMF as Maillard reaction markers. None of the powders had a content higher than 19.44 μg/g powder db (limit of quantification) and 20.16 μg/g powder db (limit of quantification) of FF and 5-HMF, respectively. Although the air inlet temperatures during SD ranged from 150 to 190 °C, these conditions did not lead to the formation of these process contaminants, especially when protein-based carriers were applied. The absence of detectable FF and 5-HMF, even at relatively high spray drying temperatures, may be attributed to the inhibitory effects of caffeic acid derivatives naturally present in *E. purpurea*, as reported in model systems [60,61]. Therefore, SD may serve as a more suitable alternative to FD for *Echinacea* extract powder production, also in terms of absence of process contaminants.

### 2.11. Free Amino Groups (OPA Assay)

The free amino groups in controls showed that in freeze-dried samples, the level of available free amino groups was lower (Table 4), which may indicate an increased protein degradation in relation to spray drying. Comparing the free amino groups results among the SD controls, it was found that increasing the inlet air temperature did not result in significant progress of the Maillard reaction [62]. When carrier-added samples were considered, it was observed that the free amino group content was reduced by, an average, 38% after FD compared to SD powders. Consistent with the free amino groups of controls, increasing the SD inlet air temperature of carrier-added samples did not correlate with a decrease in available lysine content, thus increasing the temperature in the range of 150–190 °C did not result in an acceleration of the Maillard reaction progress. The type of carrier had no significant effect on the content of N-α-acetyl-*L*-lysine in the FD powders. In the case of SD powders with an air inlet temperature of 150 and 170 °C, significantly higher free amino groups results were obtained for powders with PPI addition compared to M and M+PPI. The increased protein reactivity in powders with PPI added could be due to the higher protein content in the liquid feed mixture, affecting the nutritional value of these products.

## 3. Materials and Methods

### 3.1. Material

Approximately 10 kg of dried *E. purpurea* root was purchased from Agrest Sp. z o.o. (Lublin, Poland). The root was extracted with 70% ethanol (*v/v*) solution for 1 h at a mass to volume ratio of 1:5 (*w/w*) in 2 technical repetitions (*n* = 2). The extraction was assisted by ultrasound at a power of 0.125 W/mL and a frequency of 30 kHz generated by a Hielscher UP100H processor (Teltow, Germany) and Hielscher MS10 sonotrode (Teltow, Germany). The extract was reduced by the volume of ethanol on an evaporator under reduced pressure (RE121, Büchi, Flawil, Switzerland).

Unadulterated ethanol alcohol (≥96%) and acetate acid (≥99.5%) were purchased from Avantor Performance Material (Gliwice, Poland). Folin–Ciocalteu reagent (1.9 M/L), 2,2′-Azino-bis(3-ethylbenzothiazoline-6-sulfonic acid) (≥98%), potassium persulfate (≥99.0%), methyl 4-hydroxybenzoate (≥99.0%), furfural (≥99.0%), 5-(Hydroxymethyl)furfural, sodium hydroxide (≥99.0%), sodium dodecyl sulfate (≥99.0%), acetonitrile gradient grade and methanol gradient grade were obtained from Merck (Darmstadt, Germany). Tetrabutylammonium bromide (≥98%) was used from abcr (Karlsruhe, Germany). Ultrapure water (conductivity ≤ 4.3 µS/cm) for extraction and liquid chromatography was purified using the HLP5 system (Hydrolab, Straszyn, Poland).

### 3.2. Methods

#### 3.2.1. Drying

The *E. purpurea* root extract (15.5 ± 0.1 °Bx) divided into portions of approximately 400 g was mixed with 5% (*w*/*w*) water (control sample, C) or maltodextrin (M; Pepees S.A., Łomża, Poland), pea protein isolate (PPI; Myvegan, Manchester, United Kingdom) and a blend of maltodextrin and pea protein isolate (M+PPI) in a 1:1 (*w*/*w*) ratio (established experimentally). The control sample and the mixes with carriers were submitted for freeze-drying (FreeZone, Labconco Corp., Kansas City, MO, USA) at a chamber temperature of −60 °C and heating plate of 25 °C under reduced pressure of 65 Pa for 24 h and spray drying (Mini Spray dryer B-290 Advanced, Büchi, Flawil, Switzerland) using a two-fluid nozzle atomizer with an inside diameter of 1.5 mm with a constant air inlet temperature setting in two technical repetitions (*n* = 2). The spray drying parameters are specified in Table 5.

#### 3.2.2. Physicochemical Properties

Moisture content (*Mc*) analysis was performed in duplicate (*n* = 2) according to Michalska et al. [63] and expressed as a % of wet basis. Water activity (*a_w_*) was analyzed twice (*n* = 2) at room temperature (Water Activity Meter, 4TE, AQUA LAB, Pullman, WA, USA). Bulk density (ρb) was determined as the ratio of powder mass (m) to bulk volume (V_b_). Powder mass was measured twice (*n* = 2) in a measuring cylinder with a capacity of 10 ± 0.5 mL and expressed in kg/m^3^. Bulk density was calculated according to Equation (1):

(1) $$\rho_{b}=\frac{m}{V_{b}}$$

The true density (ρt) was calculated as the ratio of the mass of dry solids (m) to the total volume of the sample (Vs), excluding any air spaces, in accordance with Equation (2), and expressed in kg/m^3^:

(2) $$\rho_{t}=\frac{m}{V_{s}}$$

The mass of the powders was measured using an analytical balance with a precision of 0.0001 g (XA 60/220/X, Radwag, Radom, Poland). The total volume excluding air (V_s_) was determined using a HumiPyc Model 2 Gas Pycnometer (InstruQuest Inc., Coconut Creek, FL, USA).

The porosity (ε) of the powders was calculated by examining the ratio between their bulk density and true density, as described in Equation (3), according to Bhusari et al. [64].

(3) $$\varepsilon =\left(1-\frac{\rho_{b}}{\rho_{t}}\right)100\%$$

The color of the powders was determined twice (*n* = 2) in the CIE L*a*b* system (Minolta Chroma Meter CR-400 colorimeter; Minolta Co., Ltd., Osaka, Japan). The browning index (BI) of the powders was calculated based on Equations (4) and (5), according to Palou et al. [65].

(4) $$BI=\frac{\left[100(X-0.31)\right]}{0.17}$$

where X is as follows:

(5) $$X=\frac{\left(a+1.75L\right)}{\left(5.645L+a-3.012b\right)}$$

#### 3.2.3. Determination of Alkamides Content

The content of 6 alkamides was analyzed: undeca-2E, 4Z-dien-8, 10-diynoic acid isobutylamide (alkamide 1); undeca-2Z, 4E-dien-8, 10-diynoic acid isobutylamide (alkamide 2); dodeca-2E, 4Z-dien-8, 10-diynoic acid isobutylamide (alkamide 3); undeca-2E, 4Z-dien-8, 10-diynoic acid 2-methylbutylamide (alkamide 4); dodeca-2E, 4E, 8Z, 10E-tetranoic acid isobutylamide (alkamide 5); and dodeca-2E, 4E, 8Z, 10Z-tetranoic acid isobutylamide (alkamide 6). Approximately 100 mg of powder was dissolved in 10 mL of 40% acetonitrile solution (*v/v*) and vortexed (Vortex 3, IKA, Staufen im Breisgau, Germany) for 3 min. The sample solution was filtered through a 0.45 μm nylon filter (Zhejiang ALWSCI Technologies, Shaoxing, China). Chromatographic analysis was performed using a LC-2050C system (Shimadzu, Tokyo, Japan) with a PDA detector. Separation was performed on a LiChrospher RP-18 HPLC 5 µm C_18_(2) 100 Å, 250 × 4 mm chromatographic column (Merck, Darmstadt, Germany) with a 20 μL injection volume at 30 °C and a mobile phase flow rate of 1 mL/min using water and acetonitrile as mobile phases. A gradient elution of 30—70% in 47 min was used. The analysis was performed at a detector wavelength of 256 nm based on the response factor (determined from linearity against alkamides 1–6) to methyl-4-hydroxybenzoate. The determination procedures were performed twice (*n* = 2), and results were expressed in mg/g powder db.

#### 3.2.4. Determination of Total Phenolics Content (TPC)

Approximately 200 mg of powder was extracted in 6.8 mL of 50% methanol (*v/v*) followed by manual stirring for 3 min, sonication for 15 min, and incubation for 24 h at 4 °C. The extracts were taken out of the refrigerator and were re-sonicated. TPC analysis was performed using Folin–Ciocalteu’s reagent according to the method of Gao et al. [66] and modified by Horszwald and Andlauer [67] on a Synergy H1 spectrophotometer (BioTek Instruments Inc., Santa Clara, CA, USA). Two replicates (*n* = 2) were prepared and the TPC results were expressed in g gallic acid equivalent (GAE)/100 g powder db.

#### 3.2.5. Determination of Antioxidant Capacity In Vitro

The powders were extracted in the same procedure as for TPC analysis. Antioxidant capacity measured by TEAC ABTS was determined according to Re et al. [68] and FRAP assay was carried out according to Benzie and Strain, [69] with a Synergy H1 spectrophotometer (BioTek Instruments Inc., Santa Clara, CA, USA). The determination procedures were conducted twice (*n* = 2) and expressed as mmol Trolox Equivalent (TE) per 100 g of powder db.

#### 3.2.6. Quantification of Furfural (FF) and 5-Hydroxymethyl-*L*-furfural (5-HMF)

For powder diluent and mobile phase, a 30 mM sodium acetate buffer was prepared. Approximately 100 mg of powder was dissolved in 20 mL of sodium acetate buffer, vortexed (Vortex 3, IKA, Staufen im Breisgau, Germany) for 3 min and the obtained solution was filtered through a 0.45 μm nylon filter (Zhejiang ALWSCI Technologies, Shaoxing, China). Analyses were performed using a Nexera X2 liquid chromatograph (Shimadzu, Tokyo, Japan) with a PDA detector. Aliquots of 20 μL of sample were injected into a HyperClone 5 μm BDS C_8_ 130 Å 150 × 4.6 mm column (Phenomenex, Torrance, CA, USA). Isocratic elution was carried out for 7.5 min at 10 °C with the mobile phase consisting of 30 mM sodium acetate buffer at pH 4.5 and methanol in a ratio of 95:5 (*v/v*). Data from the PDA detector for FF were collected at 276 nm and for 5-HMF at 284 nm. The determination was performed twice (*n* = 2) and the results were expressed in μg/g powder db.

#### 3.2.7. Determination of Free Amino Groups (OPA Assay)

Approximately 30 mg of powder was mixed with 10 mL of 6% SDS solution. The suspension was then sonicated and vortexed (Vortex 3, IKA, Staufen im Breisgau, Germany) every 10 min for 30 min. After filtration through Whatman No. 40 paper filter, the samples were analyzed using the method described by Michalska et al. [70], with modifications for microplate application. *o*-Phthaldialdehyde (OPA) reagent was prepared by dissolving 16.4 mg of OPA in 2.5 mL of ethanol, adding 25 mL of 0.1 M borate buffer (pH 9.5), 400 µL of 10% β-mercaptoethanol, 5 mL of 20% SDS, and then diluting the solution to a final volume of 100 mL with water. Next, 50 µL of the sample was diluted 200 µL of the OPA reagent. The determination was performed on a Synergy H1 spectrophotometer (BioTek Instruments Inc., Santa Clara, CA, USA) at an excitation wavelength of 340 nm and an emission wavelength of 455 nm. The results were calculated based on the N-α-acetyl-*L*-lysine (0.1–1.8 mM) calibration curve and expressed as grams of N-α-acetyl-*L*-lysine per 100 g of powder dry basis. The sample assay procedure was performed twice (*n* = 2).

#### 3.2.8. Statistical Analysis

Statistical analyses were conducted using STATISTICA 13 software (StatSoft, Tulsa, OK, USA, version 13.3). Descriptive statistics, including average and standard deviations, were calculated. Group differences were evaluated using one-way ANOVA, followed by Tukey’s HSD post hoc test to determine significant differences, with the threshold for statistical significance set at *p* < 0.05.

## 4. Conclusions

This study demonstrated that spray drying, particularly with the use of a maltodextrin and pea protein isolate blend at inlet air temperatures between 150 and 170 °C, is an effective technique for producing *E. purpurea* root extract powders with higher alkamide content, retained total phenolic compounds, and maintained antioxidant capacity compared to freeze-drying. In this study, spray drying was shown to produce *E. purpurea* root extract powders with a more concentrated profile of bioactive compounds, particularly alkamides, compared to freeze-drying. Notably, a higher drying temperatures applied did not lead to the formation of Maillard reaction markers, indicating that the process, including liquid feed preparation and drying parameters applied, is suitable for preserving the chemical integrity of the product. To deepen the understanding of these findings, future research should focus on surface structural analyses of the powders and broaden the spectrum of monitored bioactive groups to further enhance the functional value of *E. purpurea* powders.

## Figures and Tables

**Table 1:** The moisture content (%), water activity, bulk density (kg/m^3^), true density (kg/m^3^) and porosity (%) of powders from *E. purpurea* root extract.

Drying Technique	Drying Temperature	Carrier Type	Moisture Content	Water Activity	Bulk Density	True Density	Porosity
FD	−60/25 °C	C	9.58 ± 0.06 ^a^	0.1551 ± 0.0011 ^a^	441.85 ± 4.79 ^b^	1434.18 ± 32.59 ^a^	69.18 ± 1.03 ^bc^
		M	7.00 ± 0.01 ^b^	0.0345 ± 0.0016 ^h^	437.19 ± 29.74 ^b^	1420.57 ± 16.34 ^a–c^	69.21 ± 2.45 ^bc^
		PPI	6.43 ± 0.11 ^b^	0.0360 ± 0.0011 ^h^	550.41 ± 1.05 ^a^	1370.87 ± 19.35 ^c–f^	59.84 ± 0.64 ^d^
		M+PPI	7.02 ± 0.06 ^b^	0.0350 ± 0.0001 ^h^	467.04 ± 15.40 ^b^	1394.77 ± 17.70 ^a–d^	66.52 ± 0.68 ^c^
SD	150 °C	C	0.92 ± 0.02 ^fg^	0.1115 ± 0.0026 ^c^	463.32 ± 18.56 ^b^	1428.15 ± 7.95 ^ab^	67.55 ± 1.48 ^c^
		M	2.31 ± 0.02 ^c^	0.0970 ± 0.0021 ^de^	400.78 ± 17.97 ^b–d^	1326.60 ± 7.82 ^f^	69.78 ± 1.53 ^bc^
		PPI	1.91 ± 0.19 ^c–e^	0.1370 ± 0.0014 ^b^	600.02 ± 32.59 ^a^	1407.00 ± 8.03 ^a–d^	57.36 ± 2.07 ^d^
		M+PPI	1.36 ± 0.43 ^d–f^	0.0821 ± 0.0002 ^fg^	357.23 ± 18.63 ^c–e^	1391.33 ± 7.49 ^a–e^	74.32 ± 1.48 ^ab^
	170 °C	C	0.70 ± 0.01 ^fg^	0.0996 ± 0.0013 ^d^	453.92 ± 29.97 ^b^	1416.44 ± 11.02 ^a–d^	67.94 ± 2.37 ^c^
		M	2.07 ± 0.11 ^cd^	0.0789 ± 0.0018 ^g^	277.99 ± 16.91 ^fg^	1360.42 ± 15.60 ^d–f^	79.56 ± 1.48 ^a^
		PPI	1.23 ± 0.04 ^ef^	0.1138 ± 0.0054 ^c^	599.15 ± 12.64 ^a^	1411.77 ± 15.26 ^a–d^	57.55 ± 1.35 ^d^
		M+PPI	1.70 ± 0.16 ^c–e^	0.0989 ± 0.0007 ^de^	347.81 ± 32.76 ^d–f^	1374.09 ± 7.58 ^b–e^	74.68 ± 2.52 ^ab^
	190 °C	C	0.25 ± 0.11 ^g^	0.0896 ± 0.0037 ^ef^	431.23 ± 3.49 ^b–c^	1413.41 ± 8.40 ^a–d^	69.49 ± 0.07 ^bc^
		M	0.61 ± 0.54 ^fg^	0.1010 ± 0.0006 ^d^	262.85 ± 0.98 ^g^	1257.94 ± 10.68 ^g^	79.10 ± 0.10 ^a^
		PPI	0.64 ± 0.01 ^fg^	0.1113 ± 0.0022 ^c^	570.32 ± 2.88 ^a^	1430.57 ± 8.78 ^ab^	60.13 ± 0.45 ^d^
		M+PPI	0.32 ± 0.10 ^g^	0.0869 ± 0.0044 ^fg^	318.9 ± 7.41 ^e–g^	1335.52 ± 9.56 ^ef^	76.12 ± 0.73 ^a^

FD—freeze-drying, SD—spray drying, C—control (no carrier-added samples), M—maltodextrin, PPI—pea protein isolate, M+PPI—blend composed of maltodextrin and pea protein isolate; ^a–h^—the same letter within column indicates a group that does not differ statistically significantly from each other (HSD Tukey; *p* < 0.05).

**Table 2:** The color parameters (CIEL*a*b*) and browning index (BI) of powders from *E. purpurea* root extract.

Drying Technique	Drying Temperature	Carrier Type	Color			BI
			L*	a*	b*	
FD	−60/25 °C	C	60.46 ± 1.40 ^h^	10.58 ± 0.82 ^a^	22.96 ± 1.66 ^a^	59.94 ± 6.49 ^a^
		M	75.85 ± 0.31 ^d–f^	5.52 ± 0.21 ^d^	22.02 ± 0.19 ^a–c^	39.03 ± 0.56 ^b–d^
		PPI	67.67 ± 0.85 ^g^	7.31 ± 0.16 ^b^	19.81 ± 0.63 ^de^	42.03 ± 2.04 ^bc^
		M+PPI	68.83 ± 1.20 ^g^	6.69 ± 0.21 ^bc^	20.41 ± 1.02 ^c–e^	41.72 ± 1.48 ^bc^
SD	150 °C	C	78.13 ± 0.50 ^de^	4.26 ± 0.03 ^e^	20.84 ± 0.49 ^b–d^	34.46 ± 1.09 ^de^
		M	88.05 ± 0.76 ^a^	1.45 ± 0.07 ^fg^	18.76 ± 0.25 ^e^	24.66 ± 0.64 ^f^
		PPI	73.92 ± 0.16 ^f^	5.93 ± 0.14 ^d^	23.86 ± 0.65 ^a^	44.15 ± 1.29 ^b^
		M+PPI	85.09 ± 0.89 ^bc^	2.00 ± 0.06 ^f^	19.94 ± 0.46 ^de^	27.87 ± 0.40 ^f^
	170 °C	C	78.44 ± 1.33 ^c^	4.41 ± 0.13 ^e^	22.94 ± 0.16 ^a^	38.06 ± 0.89 ^cd^
		M	88.67 ± 0.36 ^a^	1.23 ± 0.02 ^g^	18.44 ± 0.27 ^e^	23.82 ± 0.33 ^f^
		PPI	74.98 ± 0.69 ^f^	6.13 ± 0.24 ^cd^	23.00 ± 0.28 ^a^	42.00 ± 1.17 ^bc^
		M+PPI	83.05 ± 1.85 ^c^	1.70 ± 0.07 ^fg^	20.37 ± 0.40 ^c–e^	29.05 ± 0.60 ^ef^
	190 °C	C	77.98 ± 0.77 ^de^	4.55 ± 0.02 ^e^	22.67 ± 0.53 ^ab^	37.98 ± 0.68 ^cd^
		M	87.93 ± 0.47 ^a^	1.33 ± 0.08 ^fg^	18.80 ± 1.00 ^e^	24.66 ± 1.52 ^f^
		PPI	75.46 ± 1.09 ^ef^	5.89 ± 0.20 ^d^	23.72 ± 0.39 ^a^	42.82 ± 1.67 ^bc^
		M+PPI	86.06 ± 0.50 ^ab^	1.88 ± 0.07 ^fg^	20.27 ± 0.46 ^c–e^	27.88 ± 0.69 ^f^

FD—freeze-drying, SD—spray drying, C—control (no carrier-added samples), M—maltodextrin, PPI—pea protein isolate, M+PPI—blend composed of maltodextrin and pea protein isolate, BI—browning index; ^a–h^—the same letter within column indicates a group that does not differ statistically significantly from each other (HSD Tukey; *p* < 0.05).

**Table 3:** The alkamide content in powders from *E. purpurea* root extract.

Drying Technique	Drying Temperature	Carrier Type	Alkamide Content (mg/g powder db)						
			Alkamide 1	Alkamide 2	Alkamide 3	Alkamide 4	Alkamide 5	Alkamide 6	Sum of Alkamide
FD	−60/25 °C	C	0.25 ± 0.01 ^ab^	0.95 ± 0.01 ^a^	1.15 ± 0.02 ^a^	0.07 ± 0.01 ^a^	0.41 ± 0.01 ^a^	0.53 ± 0.01 ^a^	3.36 ± 0.05 ^a^
		M	0.19 ± 0.01 ^d–f^	0.71 ± 0.02 ^b-d^	0.84 ± 0.02 ^cd^	0.04 ± 0.01 ^bc^	0.28 ± 0.01 ^b-d^	0.37 ± 0.01 ^bc^	2.43 ± 0.05 ^cd^
		PPI	0.16 ± 0.01 ^f^	0.62 ± 0.02 ^d^	0.69 ± 0.03 ^e^	0.04 ± 0.01 ^c^	0.20 ± 0.01 ^d^	0.25 ± 0.02 ^de^	1.96 ± 0.07 ^f^
		M+PPI	0.18 ± 0.01 ^ef^	0.68 ± 0.04 ^b–d^	0.77 ± 0.05 ^c–e^	0.04 ± 0.01 ^bc^	0.24 ± 0.02 ^b–d^	0.31 ± 0.02 ^c–e^	2.22 ± 0.15 ^d–f^
SD	150 °C	C	0.28 ± 0.01 ^a^	0.94 ± 0.01 ^a^	1.04 ± 0.01 ^ab^	0.06 ± 0.01 ^ab^	0.29 ± 0.01 ^bc^	0.36 ± 0.01 ^bc^	2.97 ± 0.01 ^ab^
		M	0.19 ± 0.01 ^d–f^	0.63 ± 0.01 ^cd^	0.71 ± 0.02 ^de^	0.04 ± 0.01 ^c^	0.20 ± 0.01 ^cd^	0.24 ± 0.01 ^e^	2.01 ± 0.07 ^ef^
		PPI	0.21 ± 0.01 ^cd^	0.71 ± 0.03 ^b–d^	0.80 ± 0.04 ^c–e^	0.04 ± 0.01 ^bc^	0.22 ± 0.02 ^cd^	0.26 ± 0.02 ^de^	2.24 ± 0.13 ^d–f^
		M+PPI	0.25 ± 0.01 ^ab^	0.87 ± 0.02 a	1.04 ± 0.05 ^ab^	0.06 ± 0.01 ^ab^	0.32 ± 0.03 ^b^	0.37 ± 0.02 ^bc^	2.90 ± 0.14 ^b^
	170 °C	C	0.26 ± 0.02 ^ab^	0.92 ± 0.05 ^a^	1.01 ± 0.05 ^b^	0.06 ± 0.01 ^ab^	0.27 ± 0.05 ^b–d^	0.44 ± 0.08 ^ac^	2.96 ± 0.15 ^b^
		M	0.18 ± 0.01 ^d–f^	0.62 ± 0.01 ^d^	0.69 ± 0.01 ^e^	0.04 ± 0.01 ^c^	0.20 ± 0.01 ^d^	0.25 ± 0.01 ^de^	1.97 ± 0.02 ^f^
		PPI	0.19 ± 0.01 ^d–f^	0.73 ± 0.02 ^b^	0.83 ± 0.03 ^cd^	0.05 ± 0.01 ^bc^	0.24 ± 0.01 ^b–d^	0.30 ± 0.01 ^c–e^	2.34 ± 0.1 ^d–f^
		M+PPI	0.24 ± 0.01 ^bc^	0.87 ± 0.02 ^a^	0.99 ± 0.02 ^b^	0.05 ± 0.01 ^bc^	0.28 ± 0.01 ^b–d^	0.35 ± 0.01 ^b–d^	2.77 ± 0.03 ^bc^
	190 °C	C	0.19 ± 0.01 ^d–f^	0.69 ± 0.04 ^b–d^	0.71 ± 0.06 ^de^	0.04 ± 0.01 ^bc^	0.22 ± 0.05 ^cd^	0.29 ± 0.05 ^c–e^	2.13 ± 0.22 ^d–f^
		M	0.18 ± 0.01 ^d–f^	0.65 ± 0.01 ^b–d^	0.71 ± 0.01 ^de^	0.04 ± 0.01 ^c^	0.22 ± 0.01 ^cd^	0.28 ± 0.01 ^c–e^	2.07 ± 0.01 ^d–f^
		PPI	0.18 ± 0.01 ^d–f^	0.67 ± 0.01 ^b–d^	0.75 ± 0.01 ^c–e^	0.04 ± 0.01 ^bc^	0.22 ± 0.01 ^cd^	0.29 ± 0.01 ^c–e^	2.15 ± 0.01 ^d–f^
		M+PPI	0.20 ± 0.01 ^de^	0.72 ± 0.01 ^bc^	0.85 ± 0.01 ^c^	0.04 ± 0.01 ^bc^	0.26 ± 0.01 ^b–d^	0.32 ± 0.01 ^c–e^	2.41 ± 0.01 ^c–e^

FD—freeze-drying, SD—spray drying, C—control (no carrier-added samples), M—maltodextrin, PPI—pea protein isolate, M+PPI—blend composed of maltodextrin and pea protein isolate; db—dry basis; ^a–f^—the same letter within column indicates a group that does not differ statistically significantly from each other (HSD Tukey; *p* < 0.05).

**Table 4:** Total phenolics content (g GAE/100 g powder db), antioxidant capacity (mmol Trolox/100 g powder db), furfural and 5-hydroxymethyl-*L*-furfural content (μg/g powder db), free amino groups content (g N-α-acetyl-*L*-lysine/100 g powder db) of powders from *E. purpurea* root extract.

Drying Technique	Drying Temperature	Carrier Type	TPC	Antioxidant Capacity		FF	5-HMF	Free Amino Groups
				TEAC ABTS	FRAP			
FD	−60/25 °C	C	4.83 ± 0.54 ^a^	27.19 ± 1.23 ^a^	27.39 ± 0.53 ^a^	<LOQ	<LOQ	4.64 ± 0.01 ^cd^
		M	2.50 ± 0.21 ^bc^	18.88 ± 0.54 ^bc^	18.48 ± 0.77 ^b–e^	<LOQ	<LOQ	2.40 ± 0.26 ^gh^
		PPI	1.62 ± 0.16 ^d^	13.89 ± 0.82 ^e^	13.84 ± 0.27 ^h^	<LOQ	<LOQ	2.34 ± 0.11 ^h^
		M+PPI	2.18 ± 0.50 ^cd^	16.12 ± 0.93 ^de^	15.23 ± 0.69 ^gh^	<LOQ	<LOQ	2.22 ± 0.03 ^h^
SD	150 °C	C	4.83 ± 0.33 ^a^	28.91 ± 0.93 ^a^	28.19 ± 1.03 ^a^	<LOQ	<LOQ	6.34 ± 0.12 ^a^
		M	2.85 ± 0.08 ^bc^	19.41 ± 1.61 ^b^	19.44 ± 0.50 ^b–d^	<LOQ	<LOQ	3.58 ± 0.03 ^ef^
		PPI	2.37 ± 0.38 ^c^	15.35 ± 0.49 ^de^	15.40 ± 0.23 ^f–h^	<LOQ	<LOQ	4.77 ± 0.22 ^c^
		M+PPI	2.50 ± 0.15 ^bc^	17.97 ± 0.62 ^b–d^	17.61 ± 0.47 ^c–f^	<LOQ	<LOQ	3.08 ± 0.01 ^f^
	170 °C	C	4.72 ± 0.17 ^a^	29.49 ± 1.65 ^a^	28.61 ± 2.47 ^a^	<LOQ	<LOQ	5.53 ± 0.31 ^b^
		M	3.11 ± 0.27 ^b^	19.63 ± 1.62 ^b^	19.70 ± 0.42 ^bc^	<LOQ	<LOQ	3.03 ± 0.04 ^fg^
		PPI	2.20 ± 0.14 ^cd^	16.32 ± 0.50 ^c–d^	16.40 ± 0.51 ^e–g^	<LOQ	<LOQ	4.07 ± 0.02 ^de^
		M+PPI	2.34 ± 0.15 ^c^	16.50 ± 1.12 ^c–d^	16.89 ± 0.54 ^e–g^	<LOQ	<LOQ	3.10 ± 0.34 ^f^
	190 °C	C	4.62 ± 0.11 ^a^	28.37 ± 1.13 ^a^	29.17 ± 1.29 ^a^	<LOQ	<LOQ	6.61 ± 0.01 ^a^
		M	2.85 ± 0.20 ^bc^	19.56 ± 0.68 ^b^	20.07 ± 0.40 ^b^	<LOQ	<LOQ	4.33 ± 0.09 ^cd^
		PPI	2.19 ± 0.12 ^cd^	15.80 ± 0.65 ^de^	16.42 ± 0.17 ^e–g^	<LOQ	<LOQ	4.30 ± 0.08 ^cd^
		M+PPI	2.45 ± 0.11 ^bc^	17.10 ± 0.98 ^b–d^	17.40 ± 0.79 ^d–g^	<LOQ	<LOQ	3.66 ± 0.15 ^ef^

FD—freeze-drying, SD—spray drying, C—control (no carrier-added samples), M—maltodextrin, PPI—pea protein isolate, M+PPI—blend composed of maltodextrin and pea protein isolate, TPC—total phenolics content, TEAC ABTS—Trolox Equivalent Antioxidant Capacity measured by ABTS, FRAP—Ferric Reducing Antioxidant Potential, FF—furfural content (Limit of Qantification (LOQ) = 19.44 μg/g powder db), 5-HMF—5-hydroxymethyl-*L*-furfural content (LOQ = 20.16 μg/g powder db), LOQ—limit of quantification, db—dry basis; ^a–h^—the same letter within column indicates a group that does not differ statistically significantly from each other (HSD Tukey; *p* < 0.05).

**Table 5:** The parameters of the spray drying process applied for *E. purpurea* root extracts.

Carrier	Inlet Air Temperature [°C]	Outlet Air Temperature [°C]	Liquid Feed Rate [mL/min]	Gas Flow Rate [m^3^/h]
**C**	150	87 ± 1	4	35
**M**		85 ± 1	4	35
**PPI**		96 ± 1	4	35
**M+PPI**		97 ± 2	4	35
**C**	170	95 ± 1	4	35
**M**		97 ± 1	4	35
**PPI**		97 ± 1	4	35
**M+PPI**		95 ± 1	4	35
**C**	190	97 ± 2	4	35
**M**		96 ± 1	4	35
**PPI**		90 ± 1	4	35
**M+PPI**		94 ± 2	4	35

C—control (no carrier added samples), M—maltodextrin, PPI—pea protein isolate and M+PPI—blend composed of maltodextrin and pea protein isolate.

## Data Availability

The original contributions presented in this study are included in the article.
